# A Case-Control Study to Add Volumetric or Clinical Mammographic Density into the Tyrer-Cuzick Breast Cancer Risk Model

**DOI:** 10.1093/jbi/wbz006

**Published:** 2019-05-11

**Authors:** Adam R Brentnall, Wendy F Cohn, William A Knaus, Martin J Yaffe, Jack Cuzick, Jennifer A Harvey

**Affiliations:** 1 Queen Mary University of London, Centre for Cancer Prevention, Wolfson Institute of Preventive Medicine, Barts and The London School of Medicine and Dentistry, London, UK; 2 University of Virginia, Public Health Sciences, University of Virginia Health Sciences Center, Charlottesville, VA; 3 NantHealth, Inc., Culver City, CA, and University of Virginia, Public Health Sciences, University of Virginia Health Sciences Center, Charlottesville, VA; 4 Sunnybrook Health Sciences Center, Medical Biophysics, Sunnybrook Research Institute, Toronto, Ontario, Canada; 5 University of Virginia, Department of Radiology and Medical Imaging, University of Virginia Health Sciences Center, Charlottesville, VA

**Keywords:** breast cancer risk models, breast neoplasms, breast density, early detection of cancer, risk factors

## Abstract

**Background:**

Accurate breast cancer risk assessment for women attending routine screening is needed to guide screening and preventive interventions. We evaluated the accuracy of risk predictions from both visual and volumetric mammographic density combined with the Tyrer-Cuzick breast cancer risk model.

**Methods:**

A case-control study (474 patient participants and 2243 healthy control participants) of women aged 40–79 years was performed using self-reported classical risk factors. Breast density was measured by using automated volumetric software and Breast Imaging and Reporting Data System (BI-RADS) density categories. Odds ratios (95% CI) were estimated by using logistic regression, adjusted for age, demographic factors, and 10-year risk from the Tyrer-Cuzick model, for a change from the 25^th^ to 75^th^ percentile of the adjusted percent density distribution in control participants (IQ-OR).

**Results:**

After adjustment for classical risk factors in the Tyrer-Cuzick model, age, and body mass index (BMI), BI-RADS density had an IQ-OR of 1.55 (95% CI = 1.33 to 1.80) compared with 1.40 (95% CI = 1.21 to 1.60) for volumetric percent density. Fibroglandular volume (IQ-OR = 1.28, 95% CI = 1.12 to 1.47) was a weaker predictor than was BI-RADS density (P_diff_ = 0.014) or volumetric percent density (P_diff_ = 0.065). In this setting, 4.8% of women were at high risk (8% + 10-year risk), using the Tyrer-Cuzick model without density, and 7.1% (BI-RADS) compared with 6.8% (volumetric) when combined with density.

**Conclusion:**

The addition of volumetric and visual mammographic density measures to classical risk factors improves risk stratification. A combined risk could be used to guide precision medicine, through risk-adapted screening and prevention strategies.

**Key Messages**

- Clinical BI-RADS breast density and volumetric density may be used in combination with classical questionnaire risk factors to assess risk.
- Combining breast density with other risk factors increases the number of women accurately identified at high and lower risk of breast cancer.
- The Tyrer-Cuzick risk assessment tool has been updated to support using BI-RADS or volumetric breast density.

## Introduction

Breast cancer mortality rates in the USA have fallen substantially since 1990 because of early detection of the disease and better treatment (1–3). However, breast cancer remains a public health challenge: it is the most common cancer among women in the USA and worldwide (4), and generational changes in hormonal and reproductive breast cancer risk factor prevalence indicate that incidence will continue to rise. Further improvements in breast cancer screening and prevention are needed.

There is increasing interest in tailoring breast cancer screening and prevention strategies based on individual risk estimation (5–8). A variety of risk models has been developed to guide this process, and some are already recommended to determine eligibility for additional screening by magnetic resonance imaging (9–12). A clinical issue arising is that different models may provide different risk evaluations for the same woman, partly because they do not all incorporate the same comprehensive set of established risk factors, including breast density.

A comprehensive breast cancer risk model is desirable. A large body of evidence supports the role of breast density as a risk factor (13–15), but relatively few studies have empirically assessed breast density combined with a comprehensive set of other risk factors. The Gail model has been updated to include breast density, but the measure used has not been applied in clinical practice (12, 16); the same limitation is noted for an updated Tyrer-Cuzick model (17, 18). A model developed within the breast cancer surveillance consortium includes clinical mammographic density (BI-RADS density), but not a comprehensive set of risk factors (including age of affected relative, second-degree relatives, and weight) (19, 20).

The case-control study discussed in this article was designed specifically to improve risk assessment for breast cancer. The analysis reported here aimed to extend the Tyrer-Cuzick model by determining the risk associated with 2 measures of mammographic density after adjustment for the other factors in the model. Both of the mammographic density methods considered are feasible for clinical practice (BI-RADS and fully automated volumetric density). The prespecified hypothesis was that both measures of breast density would add information for breast cancer risk assessment to classical risk factors.

## Methods

### Ethical approval

This case-control study was approved by the institutional review boards at the University of Virginia and Sunnybrook Research Institute. The study was compliant with the Health Insurance Portability and Accountability Act. Patients participating on site gave written consent. Patients participating remotely through electronic media were granted waiver of consent.

### Study Design

All women 18–89 years of age diagnosed with breast cancer at a single institution between 2003 and 2013 who had a digital contralateral mammogram at the time of diagnosis were eligible as patient participants. Participant status (invasive breast cancer) was confirmed through chart review. All women without a breast cancer diagnosis but identified as having a digital mammogram between 2003 and 2008 (the more recent being at most 5 years before completing the questionnaire, and one at least 5 years before the questionnaire) were eligible as control participants. To ensure a similar age distribution, control participants were selected based on frequency matching of current age. Risk factor information was collected for patient and control participants between May 2012 and December 2013, using a self-reported electronic questionnaire that was administered in the breast imaging clinic, breast surgery clinic, or medical oncology clinic. Women who were eligible as patient participants but not seen in more than 2 years from initiation of patient recruitment were sent a letter for either survey completion by mail or online through an electronic token. Women were excluded if they had breast augmentation, prior contralateral mastectomy, or bilateral breast cancer at the time of initial diagnosis because these may affect breast density measurement. The study had sufficient power to detect breast density as a risk factor because of the larger sample size compared with those of several earlier studies on this issue (14, 15).

The public institution provides reduced fee health care based on need, such that women with greater burden of disease and low resources are frequently referred for care. Thus, we expected some differences between patient participants and control participants because control participants would mostly include women attending regular screening provided by a health plan, but patient participants might not. As a result, we considered several demographic factors for inclusion as adjustments in the analysis. These were the concentric geographical area surrounding the institution, health insurance, and whether the woman had been assessed for financial assistance, ethnicity, education, and body mass index (BMI). Age in the 5-year groups was adjusted following the study design.

Classic hormonal and reproductive risk factors included on the questionnaire were combined for adjustment using 10-year risk from the Tyrer-Cuzick (version 7.02) (3). The questionnaire information was used without modification, except for menopausal information if the mammogram preceded menopause. Accurate information on prior benign breast disease and hormone replacement therapy use was not available. Only women aged 40–79 years at mammogram were included to reflect risk assessment for women attending screening.

Full-field digital mammograms (“for processing”) DICOM files from Senographe 2000D, Senographe DS, and Senographe Essential (GE Healthcare, Chicago, IL) and Lorad Selenia and Selenia Dimensions (Hologic, Marlborough, MA) machines were retrieved. Breast density was measured using a fully automated volumetric software program (Volpara, v 1.4.5, Volpara Analytics, Wellington, New Zealand). The primary breast density value was the estimated percentage of the breast by volume occupied by fibroglandular tissue. Absolute fibroglandular and fat volume (total volume minus fibroglandular volume) were secondary predictors. For patient participants, mammograms from the contralateral breast that were taken before and closest to the time of diagnosis of breast cancer were used. For control participants, the images closest to the questionnaire were used. The mean density from all 4 mammographic views (craniocaudal and mediolateral oblique for each breast) was used for control participants, but only measurements from the contralateral side were used for patient participants. BI-RADS density category was obtained from clinical records, which were based on both breasts and the 4^th^-edition lexicon because of the time frame of the study population.

### Statistical Analyses

Weighted kappa coefficients assessed the association between volumetric and clinical BI-RADS categories. Mammographic density was incorporated into the Tyrer-Cuzick risk model by developing a measure of breast density independent from age at mammogram and BMI at the time the questionnaire was filled, and it was defined as the difference between observed and expected density. Expected density was modeled in control participants by fitting a generalized additive model (21) of natural log transformed volumetric percent density against splines for age and BMI, and the same was done for BI-RADS density by treating the categorical variables as integers from 1 (fatty) to 4 (dense).

Logistic regression was used to estimate odds ratios after adjustment for age at mammogram (5-year intervals, eg, 40–44, 45– 49, etc.), region (the immediate surrounding region [primary service area] or outlying regions, where women are less likely to obtain routine screening), and demographic factors (insurance, financial screening [ever versus never assessed], education, and ethnicity). Heterogeneity was tested using an interaction test. Tests for differences in predictive ability between 2 density measures were based on a nonparametric bootstrap test. An adjusted concordance index (mC) was used to assess discrimination further, formed by fitting a linear regression of the predictor against adjustment factors and taking the residual (22, 23).

Sensitivity analysis assessed the assumption of a linear relationship between risk and the density residual, using a generalized additive model. Interactions between density, age, and BMI were also assessed assuming a linear effect of the density residual in a logistic regression model. The proportions of participants at increased risk (greater than 8% 10-year risk) and those at decreased risk (less than 2% 10-year risk) as previously used were estimated using density combined with the Tyrer-Cuzick model.

All tests were 2 sided, and *P* < 0.05 was called significant. For analysis, we used the statistical software R version 3.4.1 and with the mgcv package (21, 24).

## Results

### Sample Characteristics

In our sample, 658 cancers were diagnosed between 2003 and 2013, following at least 1 digital mammography. We excluded 125 participants because they were deceased or lost to follow up before a questionnaire could be administered. After the exclusions shown in Figure S1, there were 474 patient participants, and 2243 control participants.

For patient participants, the index mammogram was a mean 0.5 years before diagnosis, and questionnaires were administered at a mean 3.5 years after diagnosis. The index mammogram for control participants was a mean 1.8 years before the questionnaire was administered. Detection methods used for the patient participants were mammographic screening (n = 343), clinical detection of a lump (n = 96), reporting other breast symptoms including pain and nipple discharge (n = 14), imaging other than mammography (n = 21), and unknown ( n = 3).

Patient participants were more likely than control participants were to live farther away from the institution, use Medicaid, have no health insurance, and be assessed for financial assistance, whereas, proportionally, more control participants had a higher level of education and were white (Table 1). Compared with control participants, patient participants were more likely to report nulliparity, a family history of breast cancer, early menarche, and a higher BMI (Table 1).

**Table 1.** Characteristics of the Sample

		Control	Case	OR (95% CI)
**(a) Demography adjustment factors**				
Age at mammogram (y)	Median (IQR); OR per IQR in control participants	59 (53–66)	58 (51–65)	0.90 (0.77–1.05)
Region (n, %)	Primary service *vs* outlying area	1028/2243 (46%)	146/474 (31%)	1.90 (1.54–2.35)
Health Insurance (n, %)	Insured	1528 (68%)	257 (54%)	Reference
	Medicaid	660 (29%)	198 (42%)	1.78 (1.45–2.19)
	None	55 (2%)	19 (4%)	2.05 (1.20–3.52)
Financial screening (n, %)	Ever assessed *vs* never	122/2243 (5%)	73/474 (15%)	3.16 (2.32–4.31)
Education (n, %)	Less* *vs* more education	740/2243 (33%)	231/474 (49%)	1.93 (1.58–2.36)
Ethnicity (n, %)	Not white *vs* white	207/2243 (9%)	80/474 (17%)	2.00 (1.51–2.64)
**(b) Breast cancer risk factors**				
Age at first child (y)	<20	246 (11.0%)	68 (14.3%)	0.85 (0.60–1.19)
	20–29	1212 (54.0%)	248 (52.3%)	Reference
	30+	325 (14.5%)	57 (12.0%)	1.13 (0.81–1.57)
	Nulliparous	122 (5.4%)	30 (6.3%)	1.38 (0.89–2.14)
	Unknown	338 (15.1%)	71 (15.0%)	1.19 (0.88–1.61)
Menopausal status	Pre	465 (20.7%)	107 (22.6%)	Reference
	Post	1746 (77.8%)	359 (75.7%)	1.03 (0.72–1.47)
	Unknown	32 (1.4%)	8 (1.7%)	1.02 (0.44–2.39)
Affected first-degree relatives (n, %)	None	1754 (78.3%)	353 (74.8%)	Reference
	1	458 (20.4%)	105 (22.2%)	1.19 (0.92–1.52)
	2+	29 (1.3%)	14 (3.0%)	2.48 (1.26–4.88)
Age at menarche (y)	Median (IQR)	13 (12–13)	12 (12–13)	0.92 (0.86–0.99)
	Unknown (n, %)	2 (<1%)	1 (<1%)	
Height (m)	Median (IQR)	1.63 (1.60–1.68)	1.63 (1.60–1.68)	1.05 (0.94–1.18)
	Unknown (n, %)	0 (0%)	0 (0%)	
Body mass index (kg/m^2^)	Median (IQR)	25.6 (22.6–29.9)	27.5 (23.3–32.2)	1.22 (1.08–1.37)
	Unknown (n, %)	0 (0%)	0 (0%)	
Tyrer-Cuzick risk (10y %)	Median (IQR)	3.17 (2.35–4.56)	3.29 (2.30–4.94)	1.27 (1.14–1.40)

Ref, reference category (OR = 1). *Less education includes “8th grade or less,” “Some high school,” “Completed high school,” and “Some college.”

There was significant heterogeneity in risk associated with classical factors between the primary service area, which is most representative of women attending routine screening, and outlying areas, where symptomatic women who were less likely to have private medical insurance formed a greater proportion of patient participants (*P* < 0.001, Table S1). This was reflected by a smaller difference in Tyrer-Cuzick risk assessment between patient participants and control participants in the outlying areas (Table S1) than in the subgroup that was most representative of women attending routine screening (primary service area). In the primary service area the Tyrer-Cuzick model relative risk was well calibrated (calibration coefficient from regression of predicted 10-year risk on observed risk 97% [95% CI = 58% to 135%] Table S1).

### Mammographic Density

**Figure 1.**
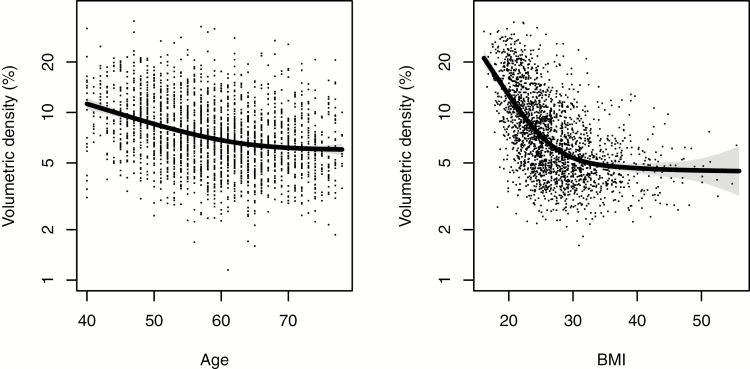
Association between volumetric percent density, age, and BMI in control participants. Points show the actual density for each woman, and the line corresponds to the smoothed expected percentage density for a woman of the given age or BMI; standard errors are shaded around the line.

Age was significantly negatively correlated with volumetric percent breast density (Spearman correlation ϱ = −0.25, Figure 1) and absolute fibroglandular volume (ϱ = −0.21), and positively correlated with fat volume (ϱ = 0.08; all *P* < 0.0001). BMI was significantly negatively correlated with percent density (ϱ = −0.56, Figure 1) and significantly positively correlated with both fibroglandular (ϱ = 0.26) and fat volume (ϱ = 0.76). Volumetric percentage density was observed to decrease both with age and BMI, but less so at the largest values, with a steeper gradient for BMI (Figure 1). BI-RADS density showed similar associations with age and BMI (Table S2).

Table 2 shows a cross tabulation of the 2 measures in patient participants and control participants, where the overall weighted κ statistic was 0.64 (95% CI = 0.62 to 0.66). There was no significant heterogeneity by region of volumetric percent density (*P* = 0.6) or BI-RADS density (*P* = 1.0).

**Table 2.** Agreement between BI-RADS and Volumetric Percent Density Grades by Patient and Control Participant Status

BI-RADS density	VPD fatty	VPD scattered	VPD het	VPD dense	Total
(a) Control participants					
1. Fatty	388 (79%)	98 (20%)	6 (1%)	0 (0%)	492 (100%)
2. Scattered	217 (25%)	445 (51%)	208 (24%)	8 (1%)	878 (100%)
3. Heterogeneous	4 (1%)	116 (17%)	434 (64%)	124 (18%)	678 (100%)
4. Dense	0 (0%)	2 (1%)	32 (16%)	161 (83%)	195 (100%)
Total	609 (27%)	661 (29%)	680 (30%)	293 (13%)	2243 (100%)
(b) Case participants	Case				
1. Fatty	59 (78%)	16 (21%)	1 (1%)	0 (0%)	76 (100%)
2. Scattered	50 (26%)	101 (53%)	35 (19%)	3 (2%)	189 (100%)
3. Heterogeneous	5 (3%)	36 (22%)	93 (57%)	29 (18%)	163 (100%)
4. Dense	0 (0%)	0 (0%)	7 (15%)	39 (85%)	46 (100%)
Total	114 (24%)	153 (32%)	136 (29%)	71 (15%)	474 (100%)

Women in the highest BI-RADS density category (9% of control participants) were 3 times (OR = 3.00, 95% CI = 1.87 to 4.81, Table 3) more likely to develop breast cancer than were those with predominately fatty breasts (22% of control participants), after adjustment for age, BMI, demographic factors, and classical questionnaire risk factors. Using volumetric density, the OR for the equivalent comparison between extremely dense (15% of control participants) and fatty categories (27% of control participants) was 2.42 (95% CI = 1.60 to 3.65). Discrimination measured by a matched concordance index (mC) was 0.58 (95% CI = 0.55 to 0.61) for BI-RADS density and 0.57 (95% CI = 0.54 to 0.59) for volumetric density. Adjusted volumetric percentage density was not a stronger predictor than BI-RADS density was (LR- 22.0 vs 31.2, *P* = 0.11).

**Table 3.** Adjusted Odds Ratios Associated with Mammographic Density

Mammographic density	Control participant	Case participant	OR (95% CI)	LR-χ^2^	*P*
(a) BI-RADS	0.0 (-0.8–0.7)	0.3 (-0.5–0.8)	1.55 (1.33–1.80)	31.2	<0.0001
1. Fatty (<25%)	492 (21.9%)	76 (16.0%)	Ref		
2. Scattered (25–50%)	878 (39.1%)	189 (39.9%)	1.86 (1.36–2.55)		
3. Heterogeneous (50–75%)	678 (30.2%)	163 (34.4%)	2.53 (1.79–3.57)		
4. Dense (>75%)	195 (8.7%)	46 (9.7%)	3.00 (1.87–4.81)		
(b) Volumetric percentage	0.0 (-0.7–0.7)	0.2 (-0.5–0.9)	1.40 (1.21–1.61)	22.0	<0.0001
1. Fatty (<4.6%)	609 (27.2%)	114 (24.1%)	Ref		
2. Scattered (4.6-<7.6%)	661 (29.5%)	153 (32.3%)	1.39 (1.04–1.85)		
3. Heterogeneous (7.6-<15.4%)	680 (30.3%)	136 (28.7%)	1.52 (1.10–2.09)		
4. Dense (15.4%+)	293 (13.1%)	71 (15.0%)	2.42 (1.60–3.65)		
Quantile 1 (least dense)	449 (20%)	75 (15.8%)	Ref		
Quantile 2	448 (20%)	80 (16.9%)	1.13 (0.79–1.61)		
Quantile 3	449 (20%)	88 (18.6%)	1.14 (0.80–1.63)		
Quantile 4	448 (20%)	95 (20.0%)	1.29 (0.91–1.82)		
Quantile 5 (most dense)	449 (20%)	136 (28.7%)	1.89 (1.36–2.62)		
(c) Volumetric glandular volume	0.0 (-0.7- 0.6)	0.1 (-0.5- 0.8)	1.26 (1.10–1.44)	11.2	0.0008
Quantile 1 (least dense)	449 (20%)	85 (17.9%)	Ref		
Quantile 2	448 (20%)	76 (16.0%)	0.86 (0.60–1.22)		
Quantile 3	449 (20%)	99 (20.9%)	1.20 (0.86–1.68)		
Quantile 4	448 (20%)	90 (19.0%)	1.01 (0.72–1.42)		
Quantile 5 (most dense)	449 (20%)	124 (26.2%)	1.61 (1.17–2.23)		
(d) Volumetric fat volume	0.0 (-0.6–0.6)	-0.1 (-0.7–0.6)	0.89 (0.79–1.01)	3.2	0.07
Premenopausal	0.1 (-0.6–0.6)	-0.4 (-0.9–0.3)	0.71 (0.55–0.93)	6.5	0.01
Postmenopausal	0.0 (-0.6–0.7)	0.0 (-0.6–0.6)	0.98 (0.85–1.14)	0.0	1.0
Quantile 1 (least fatty)	449 (20%)	113 (23.8%)	Ref		
Quantile 2	448 (20%)	90 (19.0%)	0.85 (0.61–1.17)		
Quantile 3	449 (20%)	96 (20.3%)	0.89 (0.65–1.22)		
Quantile 4	448 (20%)	97 (20.5%)	0.90 (0.66–1.23)		
Quantile 5 (most fatty)	449 (20%)	78 (16.5%)	0.70 (0.50–0.98)		

Odds ratios are adjusted for demographic factors in Table 1, Body Mass Index, and logarithm 10-year Tyrer-Cuzick risk. The median and IQR for density residuals (adjusted for age and BMI) are first given with a continuous OR per IQR in control participants; LR-χ2 (1df, P) values are based on the density residual. LR-χ2: likelihood-ratio test statistic; and volumetric percent density cutoffs are used as recommended by the manufacturer to mirror BI-RADS density, 4^th^ edition (Volpara density grades).

Absolute fibroglandular volume (IQ-OR = 1.28, 95% CI = 1.12 to 1.47, LR- = 11.2, Table 3) was a weaker predictor compared with BI-RADS density (IQ-OR from residual after adjustment for age and BMI: 1.55, 95% CI = 1.33 to 1.80, *P* = 0.014) or volumetric percentage density (IQ-OR = 1.40, 95% CI = 1.21 to 1.61, *P* = 0.065). This was partly because there was some evidence that adjusted fat volume was negatively associated with breast cancer risk, with a slightly stronger effect for premenopausal women (IQ-OR = 0.70, 95% CI = 0.54 to 0.91) compared with postmenopausal women (IQ-OR = 0.90, 95% CI = 0.79 to 1.02).

Sensitivity analysis did not reject a linear effect of the volumetric density residual on risk in the logistic model (data not shown). There was some evidence of a larger risk difference between fatty and scattered BI-RADS density than between heterogeneous and scattered BI-RADS density (Table 3), which was also observed by inspection of a nonparametric smooth estimate of the odds ratio that showed increased risk at both tails of the BI-RADS density residual (data not shown). However, the pattern was not observed in a previous study (14), so we used a linear effect, which reduced the predicted relative risk between dense and fatty BI-RADS density categories compared with fitting to each category.

Further sensitivity analysis found little evidence of interaction between density and BMI after adjustment for demographic factors and Tyrer-Cuzick 10-year risk (BI-RADS density *P* = 0.15; volumetric density *P* = 0.7), nor of an interaction with Tyrer-Cuzick risk (BI-RADS density *P* = 0.45; volumetric density *P* = 0.27). There was some evidence of attenuation in risk from density with age (BI-RADS density *P* = 0.13; volumetric density *P =* 0.03), but we did not model this further because we were concerned about overfitting.

**Figure 2.**
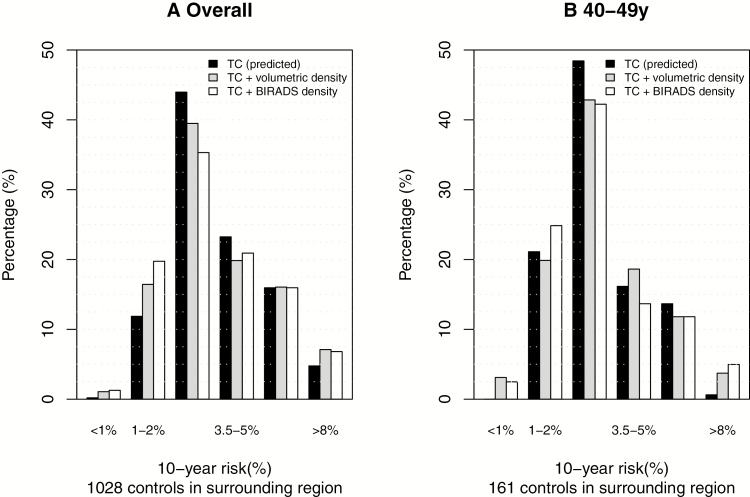
Ten-year risk distributions in the primary service area. **A:** Overall histogram of predicted absolute 10-year risk for control participants from models including density (volumetric percent and BI-RADS, based on observed risk from logistic regression applied to this study), Tyrer-Cuzick model (predicted risk), and when combined; (**B**) for women aged 40–49 years, 10-year risk distributions in the primary service area.

To assess the clinical utility of a combined risk assessment, we considered the distribution of predicted risk using women recruited as control participants from the primary service area that is most representative of women attending routine screening. The histogram in Figure 2a shows that including breast density helped us to identify accurately more high- and lower-risk women. There were 4.8% in the group using Tyrer-Cuzick without density, compared with 7.1% (BI-RADS) versus 6.8% (volumetric) with density. Similarly, 12.1% were in the lower-risk group without density, and 21.0% (BI-RADS) versus 17.5% (Volumetric) with density. The risk histogram was different by age, as seen for women age 40 to 49 years (shown in Figure 2b), where incorporating density had a greater proportional effect on the identification of those at highest risk, but fewer in total were identified.

## Discussion

This is the first study to assess the utility of automated and visual methods of mammographic density combined with the use of the Tyrer-Cuzick model in stratifying risk accurately in US women. Combining mammographic density with classical risk factors improved our ability to identify both high- and low-risk groups of women. In addition, our study focused on 2 measures of breast density that are practical for use in the USA. This is in contrast with several other studies that have evaluated the association of density with breast cancer risk using computer-assisted or visual methods to quantify the percent area of the breast occupied by breast tissue using film-screen mammograms (25–29); however, these methods are not practical for clinical use because of their requirement for substantial operator time.

Volumetric percent breast density was not more informative than BI-RADS density. However, volumetric density has some practical advantages because it is fully automated and with excellent agreement with 3-D magnetic resonance imaging (30–32). The primary advantage of using BI-RADS categories is that it is used in routine practice in the USA: more than half of the states have mandated density notification laws, requiring inclusion of breast density in mammography reports. A limitation is that BI-RADS density has substantial reader variability (33), and there have been changes in the lexicon (34).

Our findings are supported by some other studies (35) that have reported similar weighted kappa coefficients between volumetric density and BI-RADS density categories (0.57 versus 0.64 here), and similar risk associated with volumetric (mC 0.58 versus 0.57) and clinical BI-RADS density (mC 0.60 versus 0.58). It is noticeable that clinical BI-RADS density was a slightly stronger predictor in both studies. A cohort study has externally validated the adjustment for BI-RADS density that was developed in this study, and found it to be accurate for risk assessment up to 19 years after the mammogram (36).

Our study has several limitations. First, all patient participants diagnosed at the center were eligible for inclusion, and many of them were patients referred without insurance from outlying regions. Control participants differed from the overall population in ways related to geography and other socioeconomic and demographic factors. This appeared to affect the distribution of some breast cancer risk factors from the questionnaire, such as family history (Table S1), but density exhibited very little heterogeneity by geographical region. Second, risk information was obtained after cancer diagnosis, leading to the possibility of recall bias. The questionnaires were also closer on average to the mammogram for control participants than they were for patient participants because in the study design, all patient participants over the period with digital mammograms available were included, but there was a cross-sectional sample of control participants. However, the observed risks were broadly in line with those expected, so it is likely that any effect of recall bias or bias from the imbalance in time between the questionnaire and mammogram is be minimal. Third, the questionnaire was administered after the index mammogram, albeit only by 2 years on average for control participants. Fourth, there is a possible survivorship bias because some women diagnosed with breast cancer died before the questionnaire was available. This is likely to lead to an understatement of the main findings because on average, the deceased patient participants will have been diagnosed at a more advanced stage than those alive, and because density is associated with later diagnosis (masking), this bias might be expected to attenuate the predictive ability of density. Finally, it was also not possible to include patient participants who did not respond to the request to complete a questionnaire (n = 47 age 40–79 years). However, it seems unlikely that nonresponse is associated with mammographic density other than through the factors adjusted for in the analysis, such as age and demographics. Sensitivity analyses of density including the deceased and nonresponder patient participants were undertaken without adjustment for classical risk factors, but results were not materially affected (data not shown).

In conclusion, volumetric breast density and BI-RADS density add a significant contribution to the predictive power of the Tyrer-Cuzick model. The addition of breast density to risk assessment improves accurate identification of women at lower and higher risk for breast cancer, and may lead to better risk-adapted screening and prevention regimens (37).

## Funding

The work of J.H., W.F.C., W.A.K., and M.Y. was supported by Congressionally Directed Medical Research Programs (Grant number BC100474); the work of A.B. and J.C. was funded by Cancer Research UK (Grant number C569/A16891). The funding bodies had no role in the study design, analysis, interpretation of the data, or writing the manuscript.

## Supplementary Material

wbz006_Supplement_Figure_1

wbz006_Supplement_Table_1-2
