# Supplementary material for: A Case-Control Study to Add Volumetric or Clinical Mammographic Density into the Tyrer-Cuzick Breast Cancer Risk Model
Source: J Breast Imaging. 2019 May 11;1(2):99–106. doi: 10.1093/jbi/wbz006 (PMC6690422; doi:10.1093/jbi/wbz006)
Supplement: wbz006_Supplement_Figure_1 [file wbz006_supplement_figure_1.ppt]

## Slide 1
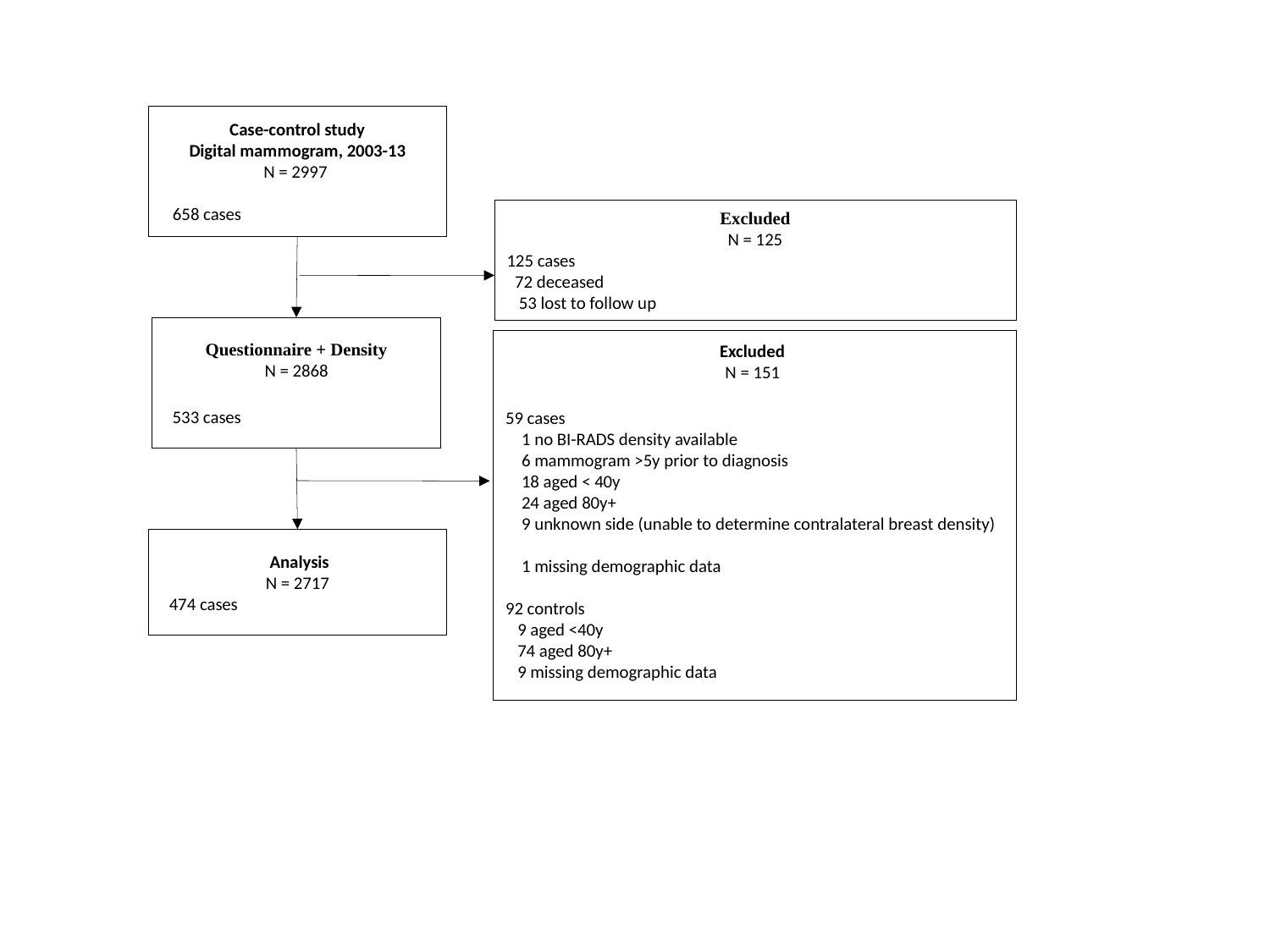

Case-control study
Digital mammogram, 2003-13
N = 2997
 658 cases
Excluded
N = 125
125 cases
 72 deceased
 53 lost to follow up
Questionnaire + Density
N = 2868
 533 cases
Excluded
N = 151
59 cases
 1 no BI-RADS density available
 6 mammogram >5y prior to diagnosis
 18 aged < 40y
 24 aged 80y+
 9 unknown side (unable to determine contralateral breast density)
 1 missing demographic data
92 controls
 9 aged <40y
 74 aged 80y+
 9 missing demographic data
 Analysis
N = 2717
 474 cases
