# Supplementary material for: A Case-Control Study to Add Volumetric or Clinical Mammographic Density into the Tyrer-Cuzick Breast Cancer Risk Model
Source: J Breast Imaging. 2019 May 11;1(2):99–106. doi: 10.1093/jbi/wbz006 (PMC6690422; doi:10.1093/jbi/wbz006)
Supplement: wbz006_Supplement_Table_1-2 [file wbz006_supplement_table_1-2.docx]

## Supplementary Tables

**Table S1.** Risk factor summary. Odds ratios adjusted for age (5-yr interval), insurance, payor status, ethnicity and education. For the continuous variables the median and inter-quartile range (IQR) are given in the case and control columns. The odds ratio is for the IQR difference in controls.

|  |  | Primary service area | | |  | Outlying regions | | | Heterogeneity |
| --- | --- | --- | --- | --- | --- | --- | --- | --- | --- |
| Risk factor | Description | Control | Case | OR (95% CI) |  | Control | Case | OR (95% CI) | P |
| Age | <20 | 70 ( 6.8%) | 13 ( 8.9%) | 0.90 (0.43-1.91) |  | 176 (14.5%) | 55 (16.8%) | 0.81 (0.55-1.19) | 0.14 |
| first | 20-29 | 538 (52.3%) | 73 (50.0%) | Ref |  | 674 (55.5%) | 175 (53.4%) | Ref |  |
| child | 30+ | 199 (19.4%) | 24 (16.4%) | 1.09 (0.65-1.83) |  | 126 (10.4%) | 33 (10.1%) | 1.33 (0.85-2.07) |  |
|  | None | 59 ( 5.7%) | 4 ( 2.7%) | 0.59 (0.20-1.69) |  | 63 ( 5.2%) | 26 ( 7.9%) | 1.83 (1.09-3.07) |  |
|  | Unknown | 162 (15.8%) | 32 (21.9%) | 1.60 (1.00-2.56) |  | 176 (14.5%) | 39 (11.9%) | 1.02 (0.68-1.52) |  |
| Menopausal | Pre | 222 (21.6%) | 36 (24.7%) | Ref |  | 243 (20.0%) | 71 (21.6%) | Ref | 0.8 |
| status | Post | 789 (76.8%) | 107 (73.3%) | 0.76 (0.39-1.48) |  | 957 (78.8%) | 252 (76.8%) | 1.11 (0.72-1.70) |  |
|  | Unknown | 17 ( 1.7%) | 3 ( 2.1%) | 0.95 (0.25-3.53) |  | 15 ( 1.2%) | 5 ( 1.5%) | 1.10 (0.36-3.37) |  |
| First | None | 809 (78.8%) | 98 (67.1%) | Ref |  | 945 (77.8%) | 255 (78.2%) | Ref | 0.031 |
| degree | 1 | 205 (20.0%) | 44 (30.1%) | 1.87 (1.26-2.78) |  | 253 (20.8%) | 61 (18.7%) | 0.94 (0.68-1.30) |  |
| relatives | 2+ | 12 (1.2%) | 4 ( 2.7%) | 2.75 (0.84-8.99) |  | 17 ( 1.4%) | 10 (3.1%) | 2.32 (1.00-5.37) |  |
|  |  |  |  |  |  |  |  |  |  |
| Menarche | yrs | 13 (12-13) | 12 (12-13) | 0.96 (0.85-1.08) |  | 13 (12-13) | 12 (12-13) | 0.90 (0.83-0.98) | 0.36 |
| Height | m | 1.63 (1.60-1.68) | 1.63 (1.60-1.70) | 1.22 (0.99-1.50) |  | 1.63 (1.57-1.68) | 1.63 (1.57-1.68) | 1.01 (0.85-1.21) | 0.15 |
| BMI | m/kg2 | 24.7 (22.0-28.7) | 26.3 (22.6-29.8) | 1.13 (0.91-1.41) |  | 26.5 (23.4-30.9) | 28.3 (24.2-33.1) | 1.21 (1.04-1.41) | 0.43 |
| Tyrer-Cuzick | 10yr % | 3.25 (2.44-4.64) | 3.80 (2.81-5.52) | 1.56 (1.31-1.86) |  | 3.10 (2.25-4.53) | 3.03 (2.09-4.48) | 1.15 (1.00-1.31) | <0.001 |

**Table S2.** BI-RADS density frequency (%) by age and BMI in controls.

| Age group | BMI | Fatty | Scattered | Heterogeneous | Dense |
| --- | --- | --- | --- | --- | --- |
| 40-49y | Normal | 0 ( 0%) | 31 (19%) | 80 (49%) | 53 (32%) |
|  | Overweight | 7 ( 8%) | 41 (46%) | 32 (36%) | 9 (10%) |
|  | Obese | 27 (28%) | 43 (44%) | 24 (25%) | 3 ( 3%) |
| 50-59y | Normal | 26 ( 7%) | 114 (32%) | 154 (43%) | 65 (18%) |
|  | Overweight | 60 (23%) | 102 (40%) | 81 (31%) | 15 ( 6%) |
|  | Obese | 72 (38%) | 79 (42%) | 36 (19%) | 1 ( 1%) |
| 60-69y | Normal | 33 (10%) | 142 (43%) | 130 (39%) | 27 ( 8%) |
|  | Overweight | 63 (27%) | 115 (49%) | 52 (22%) | 5 ( 2%) |
|  | Obese | 101 (54%) | 69 (37%) | 18 (10%) | 0 ( 0%) |
| 70-79y | Normal | 32 (20%) | 63 (40%) | 45 (29%) | 17 (11%) |
|  | Overweight | 37 (34%) | 53 (49%) | 19 (17%) | 0 ( 0%) |
|  | Obese | 34 (51%) | 26 (39%) | 7 (10%) | 0 ( 0%) |

BMI: body mass index; Normal: BMI<25 kg/m^2^; Overweight BMI 25 to <30 kg/m^2^; Obese BMI 30 kg/m^2^ or more.
